# Mastoid Abscess in a Child With Eustachian Tube Dermoid Cyst

**DOI:** 10.7759/cureus.15326

**Published:** 2021-05-30

**Authors:** Nur Afidah Mohd Zulkefli, Asma Abdullah, Noor Dina Hashim, Zara Nasseri, Thean Yean Kew

**Affiliations:** 1 Otorhinolarygology/Otology, Universiti Kebangsaan Malaysia Medical Centre, Kuala Lumpur, MYS; 2 Radiology, Universiti Kebangsaan Malaysia Medical Centre, Kuala Lumpur, MYS

**Keywords:** dermoid cyst, eustachian tube, chronic otitis media, mastoid abscess, child

## Abstract

A dermoid cyst (DC) is a benign tumor caused by inclusion errors during embryogenesis. DC of the head and neck is a well-recognized entity both clinically and histologically; however, it rarely occurs in the Eustachian tube (ET). Due to its anatomical position, significant morbidity related to middle ear dysfunction may result from ET obstruction. In this report, we present a rare case of a girl child aged two years and nine months with persistent otorrhea, who was initially diagnosed with acute otitis media with mastoiditis, along with suspicion of congenital cholesteatoma. However, high-resolution CT (HRCT) temporal and MRI of the neck revealed a DC of the ET causing left chronic otitis media (COM) with mastoid abscess. The patient underwent mastoid exploration surgery and myringotomy with grommet insertion. Although complete excision is the standard treatment modality for DC, the treatment of poorly ventilated mastoid and middle ear takes precedence over it. MRI surveillance scan is recommended in such cases.

## Introduction

Eustachian tube (ET) dermoid cyst (DC) is a rare benign developmental anomaly, resulting from embryonal disturbances during development and leads to the in-folding of the ectoderm along the normal embryonic lines of fusion [1]. Reports on DC of ET have been scarce in the literature since it was first discussed at the end of the 19^th ^century [2,3]. Based on a literature review, DC of ET is associated with benign and limited growth; however, due to its anatomical position, significant morbidity related to middle ear dysfunction may result from ET obstruction [1,4,5]. Typical clinical presentations of the condition involve recurrent refractory otorrhea, middle ear effusion, reduced hearing, and aural fullness due to mass in the ear canal and in the middle ear. Although uncommon, if DC of ET extends medially into the nasopharynx, patients may present with symptoms of upper airway obstruction [6-8]. Otoscopy findings may show mucopurulent discharge with perforated tympanic membrane (TM) or firm, pale mass in the ear canal or in the middle ear. We present a rare case of left chronic otitis media (COM) with mastoid abscess secondary to DC of ET.

## Case presentation

The patient was a girl child aged two years and nine months, who presented with persistent left otorrhea for one month. The otorrhea was foul-smelling and copious in nature, which was preceded by bouts of rhinorrhea, without fever or symptoms to suggest intracranial complications. There were no symptoms to suggest upper respiratory obstruction. She was diagnosed with acute otitis media with mastoiditis, with suspicion of congenital cholesteatoma. The child had been born full-term with a good birth weight of 3.5 kg. Both her antenatal and postnatal histories were unremarkable.

On examination, the child was found to be active with no facial asymmetry. The external ear and neck examinations were unremarkable. On otoscopy, there was foul-smelling mucopus in the left external auditory canal (EAC) with a dull, bulging TM and a pinhole perforation seen at the posteroinferior quadrant of the TM. Neither posterior wall-sagging nor keratin debris was observed.

**Figure 1:**
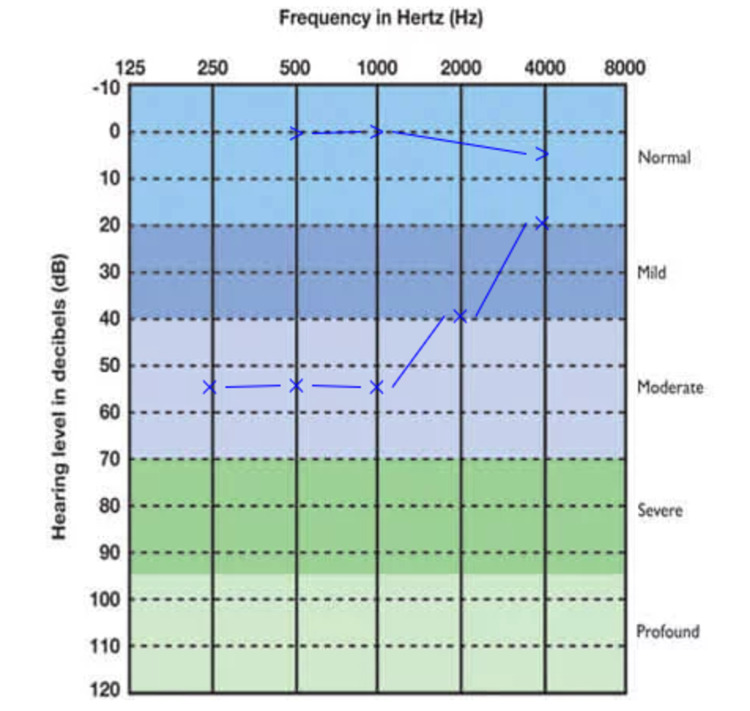
Left play audiometry Left mild to moderate conductive hearing loss was revealed

Nasoendoscopy was not performed at the time of the initial examination. Play audiometry revealed left mild to moderate conductive hearing loss (CHL) with an average air-bone gap of 36 dB (Figure 1).

**Figure 2:**
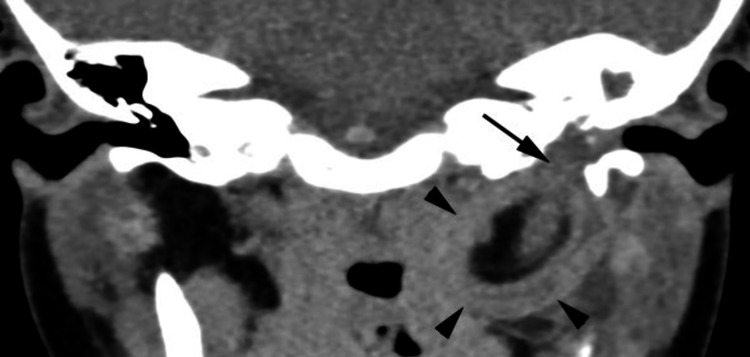
Coronal HRCT temporal (soft tissue algorithm) This CT scan depicts an opacified left middle ear and mastoid antrum with the appearance of an ovoid lesion occupying the left parapharyngeal space (arrowheads) in contrast with the normal right parapharyngeal space. The lesion is heterogeneous with predominantly fatty attenuation centrally, and a laminated soft tissue capsule. Curvilinear soft tissue traversing the fatty central matrix could represent septations. The floor of the left middle ear appears absent (arrow); this is due to the widening of the osseous Eustachian tube seen on axial images (Figure 3). The soft tissue capsule showed minimal enhancement post iodinated contrast administration (not shown) HRCT: high-resolution computed tomography

**Figure 3:**
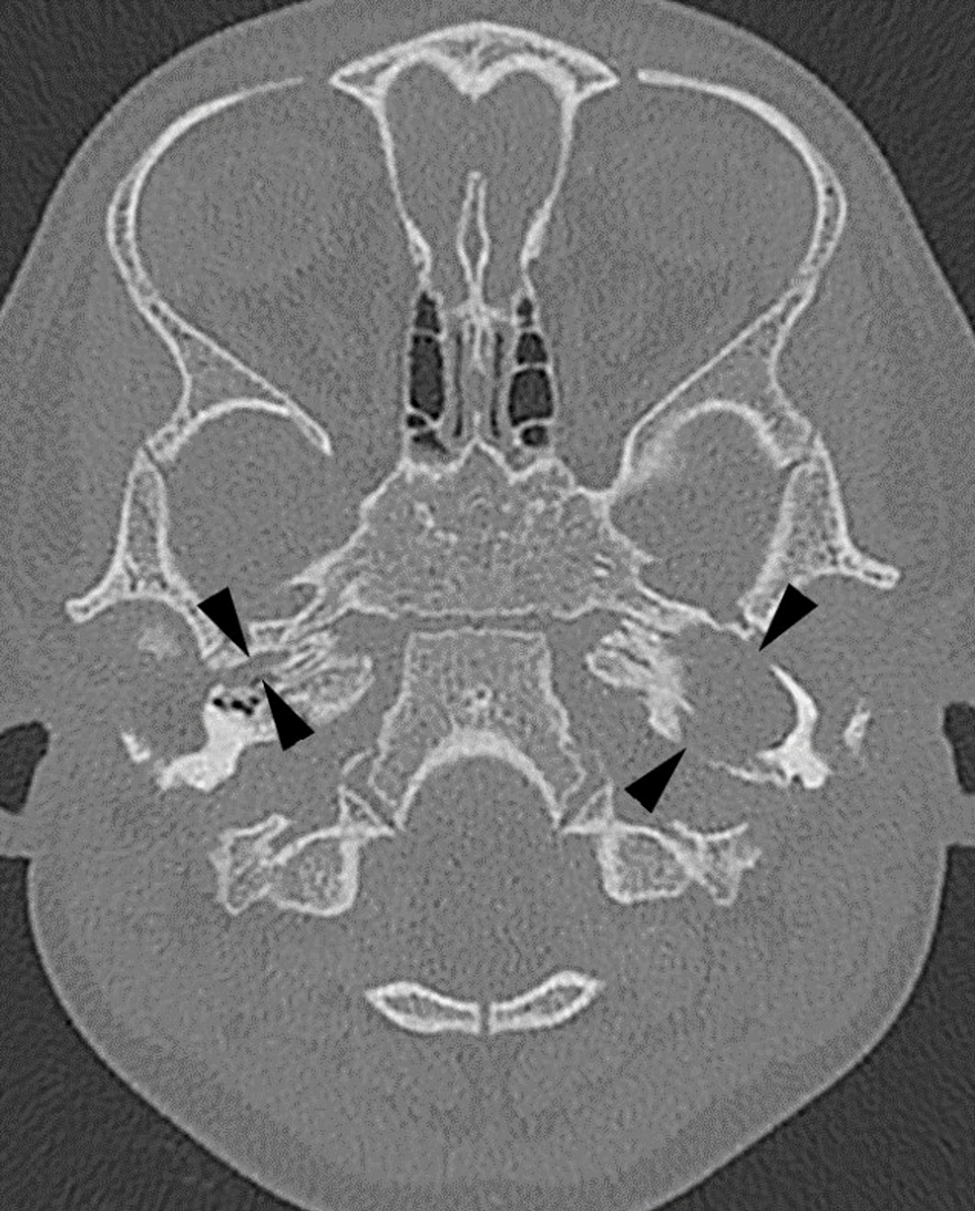
Axial HRCT temporal (bone algorithm) Axial cut at the most superior aspect of the fatty lesion displays widening of the osseous left Eustachian tube [note the normal osseous Eustachian tube on the right (black arrowheads)] HRCT: high-resolution computed tomography

The high-resolution CT (HRCT) temporal bone (Figures 2, 3) showed soft tissue occupying the bony part of left EAC, middle ear, and mastoid air cells with extension into parapharyngeal space. The floor of the left middle ear was eroded.

**Figure 4:**
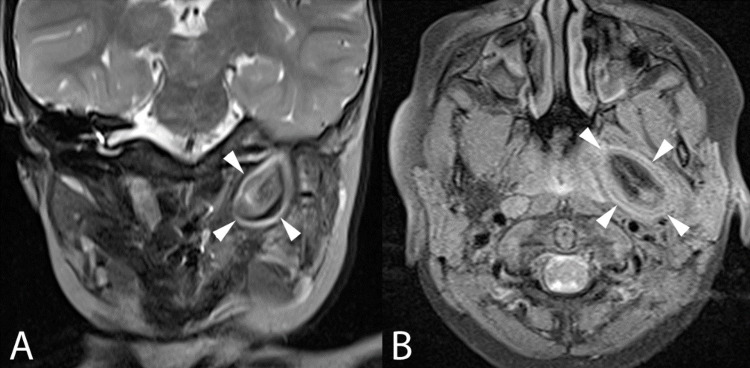
Coronal T2WI MRI (A) of the infratemporal fossa and axial fat-suppressed T1WI MRI (B) of parapharyngeal spaces These sequences depict the signal intensities of the left parapharyngeal lesion. Of note is the signal suppression of the central fatty matrix of the said lesion shown in 4B (white arrowheads), analogous to parapharyngeal and subcutaneous fat. This matrix follows the signal intensity of fat in all sequences. The laminated soft tissue capsule exhibits T2 lengthening (4A, white arrowheads). Musculature within the left masticator and prevertebral spaces are merely displaced and not infiltrated. The lesion abuts the left carotid space without definite encasement MRI: magnetic resonance imaging

MRI of the neck showed a well-encapsulated lesion within the left parapharyngeal space with heterogenous hyperintensities on T1WI (Figure 4B) and T2WI (Figure 4A), which was suppressed on fat saturation sequences with similar signal intensities in keeping with the fat content. The lesion measured 1.8 x 3.0 x 2.8 cm, laterally abutting the deep lobe of the left parotid gland with its epicenter located at the ET, extending superiorly and widening the floor of the hypotympanum, suggestive of DC of ET.

The patient underwent left canal wall up mastoid exploration and myringotomy and grommet insertion. Intraoperatively, the left mastoid was found sclerotic, with granulation tissues and pus in the mastoid cavity and middle ear. The dome of the lateral semicircular canal (SCC) was flattened. The DC was neither seen at the nasopharynx nor the middle ear. A final diagnosis of left COM with mastoid abscess secondary to DC of ET was made following pre and intraoperative evaluations.

Histopathological examination of the mastoid and middle ear lesion showed features of an acute on chronic inflammatory process. Pus culture grown did not show any bacterial growth, and tissue specimens were negative for *Mycobacterium tuberculosis* infection. At the two-week follow-up after the operation, the patient was found to be doing well with no complications. Subsequently, she was lost to follow-up due to the coronavirus disease 2019 (COVID-19) pandemic. However, upon communication with her mother via phone, the child reportedly went on to develop recurrent left otorrhea three months after the operation. Her speech is normal with no obvious reduced hearing noted by the family. She is scheduled for a repeat MRI and hearing assessment at her next follow-up.

## Discussion

DC of the head and neck accounts for 33.9% of all cases of DC, with 50% of them found to be located in the periorbital region, 25% in the oral cavity, 13% in nasal cavities, and around 12% in the neck, occipital, frontal, lip, and soft palate [1,3,6,9]. Temporal bone involvement in DC is uncommon and usually affects multiple subsites such as the middle ear, mastoid cavity, ET, and petrous apex [3,9]. With regard to DC in ET alone, we have found only 20 cases reported in the literature in English, which is an indication of its rarity. DC can be classified according to its contents into simple and complex DC. Simple DC contains only skin components such as the epidermis and dermal glands; whereas complex DC contains cartilage, bone, connective tissue, and fat [6]. McAvoy and Zuckerbraun in 1976 classified the DC of head and neck into four groups based on the inclusion sites of fusion during embryogenesis: periorbital (group 1), nasal (group 2), oral (group 3), and suprasternal, thyroid, and suboccipital (group 4) [10]. A decade later, Arcand and Abela in 1985 added on a fifth group of DC of the head and neck after reviewing seven cases of DC of ET: Eustachian tube (group 5) [11]. Kollias et al. (1995) have noted the marked predilection of DC of head and neck for the female gender, accounting for a 6:1 ratio with respect to males, and that the first presenting symptom usually occurs in early infancy. DC of ET, albeit rare, should be considered in the differential diagnosis of a child presenting with persistent otorrhea refractory to antimicrobials agents, and imaging should be promptly performed in such cases.

Differential diagnosis of benign lesions in ET includes DC, teratoma, hamartoma, and lipoblastoma. Dermoid or hairy polyps consist of only two germinal layers (mesoderm and ectoderm), which are foreign to the site. Teratoma is a true neoplasm, which consists of a mature and immature mixture of all three layers of germ cell (ectoderm, mesoderm, and endoderm), and hamartomas refer to focal disorganized overgrowth of cells and tissue found at the site of origin [5,12], while lipoblastoma is a rare lipomatous neoplasm in infants and young children, arising from embryonic white fat comprising adipocytes and lipoblasts [13]. Diagnosis of DC can be reached based on the clinical appearance of a whitish-greyish, pedunculated, or polypoidal mass covered by skin with or without hair, with the histological confirmation of a lesion composed of the dermis with skin appendages and fibrofatty material contents [1]. However, due to the unique and hidden position of the ET, visualization of a well-circumscribed fatty tumor on radiological imaging helps tremendously in making the diagnosis.

The treatment for DC of ET is often surgical resection. However, based on a literature review, the surgical resection is usually performed in children presenting with airway obstruction caused by DC of ET extending into the nasopharynx. Other cases are managed in phases, with transmastoid and nasoendoscopy approaches including neck exploration depending on the size and extension of the DC [3-7]. In this case, mastoid exploration and drainage were performed for COM with mastoid abscess. The DC was localized in the ET and was not extending into the middle ear or nasopharynx, a finding supported by HPE, which reported inflammatory tissue only in the middle ear and mastoid air cells. Therefore, we planned for surveillance MRI and hearing assessment due to the condition's benign nature, with complete surgical excision planned to be conducted in a time frame of six months to one year, depending on the patient's condition.

## Conclusions

Patients with persistent refractory otorrhea require an urgent HRCT temporal to obtain a correct diagnosis. In cases of sequelae of poor middle ear ventilation like in our patient, a multidisciplinary discussion involving the neuro-otologist, radiologist, and pediatric teams is imperative in achieving the correct diagnosis and deciding on the proper management. Treatment of the poorly ventilated mastoid and middle ear takes precedence over complete surgical excision of the tumor. MRI surveillance and preservation of function are advisable in cases of localized DC of ET without extension to the nasopharynx or middle ear.
